# The Redox-Sensitive Na/K-ATPase Signaling in Uremic Cardiomyopathy

**DOI:** 10.3390/ijms21041256

**Published:** 2020-02-13

**Authors:** Jiang Liu, Ying Nie, Muhammad Chaudhry, Fang Bai, Justin Chuang, Komal Sodhi, Joseph I. Shapiro

**Affiliations:** 1 Department of Biomedical Sciences, Joan C. Edwards School of Medicine, Marshall University, Huntington, WV 25755, USA; niey@marshall.edu (Y.N.); chaudhry@marshall.edu (M.C.); baif@marshall.edu (F.B.); 2 Department of Medicine, Joan C. Edwards School of Medicine, Marshall University, Huntington, WV 25755, USA; chuang@marshall.edu (J.C.); sodhi@marshall.edu (K.S.); shapiroj@marshall.edu (J.I.S.)

**Keywords:** Na/K-ATPase, signaling, c-Src. ROS, partial (5/6th) nephrectomy, uremic cardiomyopathy

## Abstract

In recent years, Na/K-ATPase signaling has been implicated in different physiological and pathophysiological conditions, including cardiac hypertrophy and uremic cardiomyopathy. Cardiotonic steroids (CTS), specific ligands of Na/K-ATPase, regulate its enzymatic activity (at higher concentrations) and signaling function (at lower concentrations without significantly affecting its enzymatic activity) and increase reactive oxygen species (ROS) generation. On the other hand, an increase in ROS alone also regulates the Na/K-ATPase enzymatic activity and signaling function. We termed this phenomenon the Na/K-ATPase-mediated oxidant-amplification loop, in which oxidative stress regulates both the Na/K-ATPase activity and signaling. Most recently, we also demonstrated that this amplification loop is involved in the development of uremic cardiomyopathy. This review aims to evaluate the redox-sensitive Na/K-ATPase-mediated oxidant amplification loop and uremic cardiomyopathy.

## 1. Introduction

For 200 years, digitalis-like drugs have been used to treat heart failure. This is based on the facts that digitalis-like drugs cause an inotropic effect by coupling the partial inhibition of the Na/K-ATPase ion-exchange activity (increases intracellular sodium concentration, [Na^+^]_i_) with Na^+^/Ca^2+^ exchanger (NCX) (increases intracellular calcium concentration, [Ca^2+^]_i_) to increase cardiac contractility. However, further studies were unable to directly link ouabain-mediated gene regulation effects to intracellular ionic change or the homeostasis of sodium and potassium. Later, it was demonstrated that digitalis-like drugs not only cause intracellular ionic changes but also stimulate transcriptional upregulation of several marker genes including Na/K-ATPase itself, as well as cardiac hypertrophy and cardiac fibrosis through increased cell growth and protein synthesis.

The recently discovered and appreciated Na/K-ATPase signaling function sheds new light on the understanding of its regulatory mechanism of cardiac function. The ligands of Na/K-ATPase, cardiotonic steroids (CTSs, also known as endogenous digitalis-like substances which are the base of digitalis-like drugs) have been classified as a new class of hormones, making Na/K-ATPase a potential therapeutic target [1,2,3,4].

## 2. Oxidative Stress and Oxidative Modification in Regulation of Na/K-ATPase Activity

The redox sensitivity of Na/K-ATPase has been observed in a wide range of animal species, tissues, and cell types. Increases in reactive oxygen species (ROS) and/or reactive nitrogen species (RNS) can oxidize the Na/K-ATPase α/β subunits and its independent regulator FXYD proteins, including S-glutathionylation, S-nitrosylation, phosphorylation, and carbonylation. These oxidative modifications inhibit the Na/K-ATPase activity and promote its degradation in different cell types, including cardiac myocytes, vascular smooth muscle cells, and renal proximal tubular cells, amongst others [5,6,7,8,9,10,11,12,13,14,15,16,17,18,19,20,21,22,23,24,25]. It also appears that the oxidative modifications of Na/K-ATPase are reversible [15,17,21], which means the Na/K-ATPase activity can be regulated in a redox-sensitive manner. In cardiac myocytes and the pig kidney, oxidative stress induces glutathionylation of the Na/K-ATPase β1 subunit. In the meantime, FXYD proteins can reverse oxidative stress-induced inhibition of Na/K-ATPase activity by the deglutathionylation of the β1 subunit [15].

In the oxidative modifications of Na/K-ATPase, S-glutathionylation is well documented and reviewed. S-glutathionylation of cysteine residue(s) of the Na/K-ATPase α1/β1 subunit may reduce α1/β1 association, cause conformational change, and block the α1 subunit’s intracellular ATP-binding site, leading to the inhibition of its activity [15,26,27].

In rat cerebellar granule cells, Na/K-ATPase is redox-sensitive with an "optimal redox potential range," where ROS levels out of this “optimal range” inhibit the Na/K-ATPase activity [28]. In the dog kidney, the oxidative modification of Na/K-ATPase by H_2_O_2_ also reduces cellular sulfhydryl (SH) groups [29] and results in the oligomeric structure of Na/K-ATPase [30]. The number, location, and accessibility of SH groups in Na/K-ATPase may determine the enzyme’s oxidative stability and redox sensitivity [17,31,32]. The oxidative modification of Na/K-ATPase can lead to conformational and functional changes [5,6,7,8,12,13,15,16,20,23,24,26,32,33,34,35].

## 3. The Na/K-ATPase Signaling Function is Redox-Sensitive

The signaling functions of Na/K-ATPase have been demonstrated in different cell types (such as neonatal and adult myocytes and the renal proximal tubule) as well as in different in vivo animal models [36,37,38,39,40]. As specific ligands and inhibitors of Na/K-ATPase, cardiotonic steroids (CTSs, also known as digitalis-like substances) belong to a group of structure-similar compounds, including plant-derived digitalis substances such as digoxin and ouabain, and vertebrate-derived aglycones such as bufalin and marinobufagenin (MBG). Endogenous CTSs were defined as a class of steroid hormones (1) that showed different functions [2,3,4,41,42]. Endogenously identified ouabain and MBG were widely used in the field to study the Na/K-ATPase and its signaling functions.

Ouabain-induced increases in [Ca^2+^]_i_ are also involved in ouabain-stimulated Na/K-ATPase signaling. In renal epithelial cells, low doses of ouabain induce regular, low-frequency [Ca^2+^]_i_ oscillations that lead to NF-kB activation. This process does not depend on the partial inhibition of Na/K-ATPase using low extracellular K^+^ and the depolarization of cells, but depends on the ouabain-stimulated activation of tyrosine kinase c-Src and phospholipase C–γ (PLC-γ) and the formation of Na/K-ATPase/inositol 1,4,5-trisphosphate receptors (IP3Rs) signaling micro-domain, involving the α1 N-terminus motif LKK [43,44,45,46]. On the other hand, the lowering of extracellular K^+^ can activate protein kinases and raise [Ca^2+^]_i_ in cardiac myocytes [47]. Since the effects of ouabain on c-Src phosphorylation are independent of changes in intracellular ion concentrations [38,47,48,49], it seems that the ouabain-induced inhibition of Na/K-ATPase enzymatic activity (at high ouabain concentration) and activation of c-Src-dependent signaling (at low ouabain concentration) are, at least partially, two separate regulatory mechanisms.

In isolated rat (neonatal and/or adult) cardiac myocytes, ouabain-stimulated Na/K-ATPase signaling increases ROS generation [34,38,40,50]. In isolated neonatal myocytes, ouabain-induced generation of ROS is largely prevented by overexpression of a dominant negative Ras, suggesting an ouabain-stimulated, Ras-dependent increase in ROS [38]. While the activation of Na/K-ATPase signaling by its ligands increases ROS, increases in ROS alone also activate the Na/K-ATPase signaling. In cultured cardiac myocytes, exogenously added glucose oxidase (which generates a sustained nontoxic low level of H_2_O_2_ by consuming glucose) activate the Na/K-ATPase signaling, which is sufficient to stimulate protein synthesis, cell enlargement, and the expression of several hypertrophic marker genes [34].

Ouabain also stimulates the Na/K-ATPase signaling and ROS generation in other cell types, such as renal proximal tubules [21,22,51,52,53,54]. In renal proximal tubule LLC-PK1 cells, Na/K-ATPase is involved in the glucose-oxidase-stimulated activation of the c-Src/ERK1/2 pathway [21,51], and a basal ROS level is required to initiate the ouabain-stimulated Na/K-ATPase signaling and protein carbonylation of two amino acid residues in the actuator domain of the Na/K-ATPase α1 subunit [21,22]. Moreover, infusion of MBG causes systemic ROS generation and protein oxidation, which is linked to uremic cardiomyopathy in experimental animals [55,56].

Both c-Src and caveolin-1 (structural protein of caveolae) are critical in the Na/K-ATPase/c-Src signaling. These two proteins are not only redox-sensitive but also critical in the redox-signaling platform formation [57,58,59,60,61,62]. As mentioned before, a basal level of ROS is required for the ouabain-stimulated Na/K-ATPase/c-Src signaling activation, protein carbonylation, and trafficking of Na/K-ATPase and NHE3 [21,22]. These observations are in agreement with the observations in clinical trials with antioxidant supplements in that the beneficial effect is controversial and not seen in most clinical trials [63,64,65]. The balance of the redox status, within a physiological range, may be critical in order to maintain beneficial ROS signaling.

Comparing wild-type (WT) mice with caveolin-1 knockout (cav-1 KO) mice, depletion of caveolin-1 does not affect the total or plasma membrane abundance of Na/K-ATPase α1 isoform in cardiac fibroblasts [66], adult cardiomyocytes, and left ventricle lysates [67]. In isolated cardiac fibroblasts, depletion of caveolin-1 increases the Na/K-ATPase ion-exchange activity as well as basal activities of Src and ERK1/2, but it also suppresses ouabain-induced signal transduction, growth, and collagen production [66]. In isolated adult cardiomyocytes, the ouabain-stimulated activation of phosphoinositide 3-kinase (PI3K)-α/Akt and ERK1/2, interaction of Na/K-ATPase with caveolin-3 and PI3K-α, and hypertrophic growth were significantly reduced in cav-1 KO mice, compared to WT mice [67]. Interestingly, in both WT and cav-1 KO mice, the transient infusion of ouabain induces similar positive inotropy in vivo*,* and ouabain also causes similar dose-dependent contractility in isolated working hearts [67]. The data suggest differential roles of cav-1 in the regulation of ouabain signaling and contractility.

It is worth noting that ouabain also activates the PI3K-α/Akt/β-GSK/mTOR (mammalian target of rapamycin (mTOR)) pathway that leads to physiological hypertrophy in cultured adult cardiomyocytes that is different from pathological hypertrophy [68,69,70].

## 4. The Redox-Sensitive Na/K-ATPase Signaling and Na/K-ATPase Signaling-Mediated Oxidant Amplification Loop

**Figure 1:**
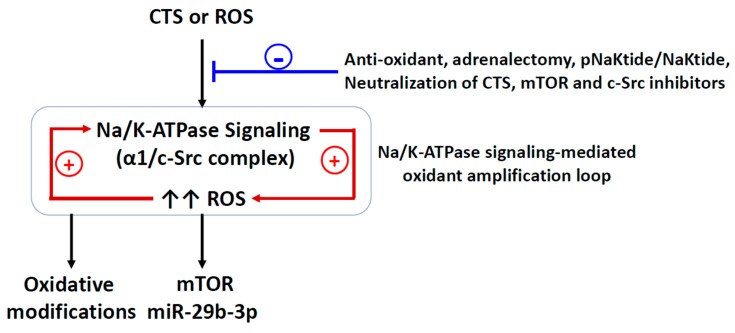
Schematic illustration of the concept of the Na/K-ATPase signaling-mediated oxidant-amplification loop. **+**, stimulating effect; **-** attenuating effect; CTS, cardiotonic steroids; ROS, reactive oxygen species; α1, Na/K-ATPase α1 subunit; mTOR, the mammalian target of rapamycin; miR-29b-3p, microRNA-29b-3p.

The early studies of the Na/K-ATPase signaling function on cardiac hypertrophy were mostly performed with CTSs, especially ouabain, at low doses that did not cause significant changes in intracellular Na^+^ concentration. Interestingly, ouabain-stimulated Na/K-ATPase signaling also increased ROS generation that was involved in the signaling function, which was independent of changes in intracellular Ca^2+^ and Na^+^ concentrations, but dependent on Ras activation [38,50]. This leads to the question of whether the Na/K-ATPase signaling could be activated by ROS alone, since oxidative modifications are able to induce conformational changes which can lead to the Na/K-ATPase α1 subunit-bound c-Src activation. A bolus of hydrogen peroxide (H_2_O_2_) or glucose oxidase (to produce H_2_O_2_) stimulates the Na/K-ATPase signaling in LLC-PK1 cells [21,22,51] and cardiac myocytes [34], while glucose oxidase stimulates the Na/K-ATPase signaling and direct protein carbonylation (Pro222 and Thr224) of the Na/K-ATPase α1 subunit that favors an E-2P conformation of Na/K-ATPase [21,22]. A single mutation of Pro222 (to alanine) and pretreatment with N-acetyl cysteine (NAC) or vitamin E disrupt ouabain- or glucose-oxidase-induced Na/K-ATPase/Src signaling and protein carboxylation [21,22]. As discussed above, the Na/K-ATPase activity and oxidative modifications could be reversibly regulated [15,16,21,22], and the partners of the Na/K-ATPase signaling, c-Src and caveolin are also redox-sensitive and critical in the redox-signaling platform formation. These observations indicate a feed-forward, redox-sensitive Na/K-ATPase signaling-mediated oxidant amplification loop stimulated by the activation of the Na/K-ATPase signaling, i.e., activation of the Na/K-ATPase signaling (either by ouabain or ROS) generates more ROS, which in turn, further activates the signaling [71] (Figure 1). This amplification loop may play an important role in overall redox regulation. Even though the Na/K-ATPase signaling-mediated oxidant amplification loop was established in the renal proximal tubule cell, the similarity of the Na/K-ATPase signaling function in both cardiac myocytes and renal proximal tubule cells suggests that this amplification loop might be shared in both cell types. However, this positive feedback mechanism might chronically desensitize the signaling function and reduce the Na/K-ATPase ion-transport capability by stimulating Na/K-ATPase/c-Src endocytosis [72,73,74].

## 5. The Na/K-ATPase Signaling and Oxidative Stress in Uremic Cardiomyopathy

Even though it is still not fully understood, clinical evidence supports the existence of a cardio-renal syndrome (worsened cardiac function leads to renal dysfunction) or reno-cardiac syndrome (worsened renal function leads to cardiac dysfunction), in which dysfunction of either the heart or the kidney can lead to pathological changes in both, increased mortality, and comorbidities [75,76,77]. One example is the development of uremic cardiomyopathy promoted by chronic kidney disease or end-stage renal disease, which has been an increased risk factor of cardiovascular disease and mortality. Uremic cardiomyopathy is characterized by diastolic dysfunction, left ventricular hypertrophy, and fibrosis, and is accompanied by deterioration in left ventricular systolic function and atrial myopathy. Recent studies indicate that endogenous CTSs- and uremic-toxins-induced oxidative stress may play an important role in the uremic cardiomyopathy development, including cardiac hypertrophy and cardiac fibrosis [55,78,79,80,81,82,83,84,85,86,87,88].

CTS-stimulated Na/K-ATPase signaling induces cardiac and renal fibrosis that can be prevented by ROS scavenging [38,55,89,90]. In the kidney and heart, the central role of CTSs in the development of fibrosis has been demonstrated in both in vivo animal models and in vitro cell culture treated with CTSs. Compared to age- and gender-matched healthy controls, in cardiac myocytes isolated from Sprague-Dawley rats, uremic serum samples (collected from end-stage renal disease patients with left ventricular hypertrophy and diastolic dysfunction) not only inhibited the Na/K-ATPase activity but also increased contractility and calcium cycling in cardiac myocytes, which can be attenuated by neutralization of the uremic serum samples with Digibind (an antibody fragment against digoxin) [91]. The partial (5/6^th^) nephrectomy model (PNx, a well-documented experimental renal failure model) shows elevated circulating levels of MBG, uremic toxins, systemic oxidant stress, and cardiac fibrosis in both rat and mouse models [55,87,90,92]. Active immunization against MBG, administration of an antibody against MBG, or reduction of MBG production by adrenalectomy substantially attenuate PNx- and MBG-infusion-mediated cardiac fibrosis and oxidant stress, an effect that is independent of blood pressure changes [55,90,93].

In rat cardiac and renal fibroblasts, MBG stimulated a PLC-dependent translocation of PKC-δ to nucleus, resulting in the phosphorylation and degradation of transcription factor Friend leukemia integration-1 (Fli-1), as well as procollagen expression [94]. Both CTS-induced Na/K-ATPase signaling and ROS generation are involved in the development of cardiac and renal fibrosis since CTS-stimulated signaling and fibrosis could be attenuated by ROS scavenging, c-Src inhibition, and competitive inhibition of CTS binding to Na/K-ATPase by spironolactone and canrenone [55,90,95,96]. In LLC-PK1 cells, MBG also stimulates the transcription factor Snail expression, leading to upregulation of collagen I, fibronectin, and vimentin expression, which is associated with epithelial-to-mesenchymal transition (EMT) during renal fibrosis [97].

Patients with chronic kidney disease (CKD) showed higher plasma MBG level compared to healthy people, and PNx rats also showed an increase in the plasma MBG level [82,90]. In Sprague-Dawley rats, PNx or MBG infusion increased plasma MBG levels, blood pressure and heart size, impaired diastolic function, and caused cardiac fibrosis. Interestingly, immunization against MBG prior to PNx surgery and concomitant PNx and adrenalectomy showed similar blood pressure as PNx but fewer changes in cardiac function and fibrosis. In cultured cardiac fibroblasts, MBG induced increases in procollagen-1 expression that could be prevented by the administration of inhibitors of tyrosine phosphorylation, c-Src activation, EGFR transactivation, and NAC [90]. Administration of a monoclonal antibody against MBG (clone 3E9) 5 weeks post-PNx attenuated PNx-mediated uremic cardiomyopathy, including increased cardiac protein carbonylation, cardiac hypertrophy, collagen-1 expression, and a reduced Fli-1 expression [55,93]. In ex vivo study, the same 3E9 antibody restored Na/K-ATPase in erythrocytes isolated from patients with CKD but did not affect Na/K-ATPase from control subjects [82]. Furthermore, PNx-mediated cardiomyopathy could be attributed to the Na/K-ATPase signaling function. The Na/K-ATPase signaling-linked mTOR pathway is involved in the cardiac fibrosis development. Compared to sham, Sprague-Dawley rats with PNx surgery or MBG infusion showed a marked increase in plasma MBG levels, hypertension, and cardiac fibrosis. Rapamycin (a mTORC1 inhibitor) treatment reduced circulating MBG levels in PNx animals, while Rapamycin treatment in combination with MBG infusion significantly attenuated cardiac fibrosis [98].

The Na/K-ATPase signaling is an important mediator in CKD that regulates microRNA (miR)-29b-3p (an anti-fibrotic microRNA that directly targets the collagen mRNA) expression and cardiac fibrosis. By using Na/K-ATPase α1 heterozygote knockout mice (α1+/−) that exhibited a reduction in the Na/K-ATPase α1 subunit and deficiency in the Na/K-ATPase signaling, α1+/− itself potentiated MBG-induced cardiac myocyte apoptosis and cardiac dilation, compared to wild-type (WT) mice [99]. This might be attributed to the MBG-activated c- Src/Akt/mTOR signaling pathway in isolated WT myocytes, but not in α1+/− myocytes [99]. In cardiac tissue from rats subjected to MBG infusion or PNx, miR-29b-3p expression was consistently reduced in both PNx and MBG-infused animals. Comparing to the control, treatment of primary cultures of adult rat cardiac fibroblasts with MBG induced significant increases in the fibrosis marker and collagen expression, accompanying a concomitant decrease in the miR-29b-3p expression [100]. Further study showed that PNx significantly reduces miR-29b-3p expression in the heart tissue by the activation of c-Src and NFκB signaling in WT mice, but fails to affect the miR-29b-3p expression and Src and NFκB signaling in the α1+/- mice [101].

Among many risk factors, elevated oxidative stress is a significant contributor to inducing cell proliferation, cardiac hypertrophy, and collagen synthesis [50,55,87,90,102,103]. Elevated circulating CTS levels also produce similar phenotypes both in vitro and in vivo [55,88,104,105]. In cultured cardiomyocytes, the CTS treatment stimulates ROS generation and NF-κB activation which could be blocked by pretreatment with antioxidants [50]. CKD patients tend to have increased circulating CTS [82,83] and dilated cardiomyopathy patients tend to have a decreased cardiac Na/K-ATPase expression [106,107]. In an experimental animal model of PNx and MBG infusion, similar uremic cardiomyopathy phenotypes, such as elevated circulating MBG, cardiac hypertrophy, impaired cardiac function, and cardiac fibrosis, were observed [55,90,108].

**Figure 2:**
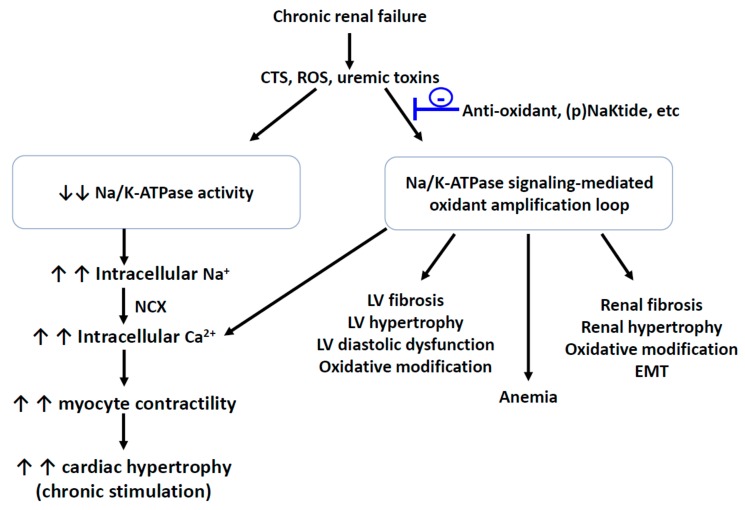
Schematic illustration of the proposed causes and consequences of the Na/K-ATPase signaling-mediated oxidant amplification loop. **-** attenuating effect; CTS, cardiotonic steroids; ROS, reactive oxygen species; NCX, sodium/calcium exchanger; EMT, epithelial-to-mesenchymal transition.

In renal proximal tubule cells, a Na/K-ATPase/Src/ROS feed-forward oxidant-amplification loop, which was involved in the Na/K-ATPase signaling activation and oxidative stress, was demonstrated [21,22]. This oxidant amplification loop was also demonstrated in vivo in the development of uremic cardiomyopathy and provided a potential drug candidate for uremic cardiomyopathy [87]. This observation, using a pole ligation PNx mouse model [109], showed PNx increased c-Src/ERK1/2 phosphorylation along with renal dysfunction, cardiac hypertrophy, cardiac fibrosis, and increased protein carbonylation in the left ventricle. Administration of pNaKtide, an antagonist of the Na/K-ATPase/Src signaling that binds to the c-Src kinase domain [110,111,112], attenuated physiological, morphological, and biochemical alterations of uremic cardiomyopathy. Interestingly, blockage of this oxidant amplification loop with pNaKtide, but not induction of HO-1, appeared to ameliorate anemia in PNx animals. Taken together, it indicates that the oxidant amplification loop is important for the development of uremic cardiomyopathy (Figure 2). Moreover, this oxidant amplification loop has also been demonstrated to affect other pathophysiological alterations in other types of animal models including the Western diet, obesity, ageing, steatohepatitis, atherosclerosis, and adipogenesis [113,114,115]. In pre-adipocyte 3T3-L1 cells and MSC-derived adipocytes, uremic toxin indoxyl sulfate and p-cresol sulfate can stimulate the Na/K-ATPase signaling and the oxidant amplification loop [116], suggesting that this oxidant amplification loop is not limited to one cell type. One of the uremic toxins, asymmetric dimethylarginine (ADMA), is a risk marker for endothelial dysfunction and cardiovascular disease by regulating nitric oxide (NO) generation in disease states. ADMA is a potent competitive inhibitor of nitric oxide synthases and its circulating concentration is increased in the failing kidney(s). An increase in circulating ADMA can lead to reduced heart rate and cardiac output, as well as left ventricular hypertrophy. Thiol containing nitric oxide-derivatives were found to inhibit the brain and kidney Na/K-ATPase activity [26,117]. Moreover, thiol modification can also regulate the interaction between the c-Src and α1 subunit. Cys 458 and 459 form the interaction interface between the α1 subunit and Src kinase and binding of glutathione to Cys 458 and 459 disrupts the interaction [118]. It will be of interest to investigate the effect(s) of ADMA on Na/K-ATPase activity and signaling in the future since it is still not well-understood.

## 6. Implications and the Possible Role of (p)Naktide

It appears that the Na/K-ATPase signaling-mediated oxidant amplification loop contributes to the development of PNx-mediated uremic cardiomyopathy, as well as other pathophysiological changes. Controlling this amplification loop within a physiological range might maintain a beneficial ROS signaling and systemic redox status, making it a promising therapeutic target.

It is worth mentioning that the (p)NaKtide might be a promising peptide for the regulation of the Na/K-ATPase signaling. The binding status of the Na/K-ATPase α1 and c-Src is the centerpiece of the Na/K-ATPase signaling, in which the α1 subunit provides the ligand-binding sites and the α1-associated c-Src provides the kinase moiety. It was demonstrated that the CD2 and ND1 segments of the α1 subunit bind to the SH2 domain and tyrosine kinase domain (KD) of c-Src through a two-pair domain binding. NaKtide was developed from the 20-amino acid sequence (SATWLALSRIAGLCNRAVFQ) of the α1 subunit ND1 segment that targets the Na/K-ATPase/c-Src signaling receptor complex and inhibits the Na/K-ATPase signaling function. pNaKtide was further developed by adding a 13-amino acid HIV-TAT leading sequence (GRKKRRQRRRPPQSATWLALSRIAGLCNRAVFQ) to facilitate its cellular permeation. This TAT leading sequence makes the pNaKtide cell membrane permeable and keeps the pNaKtide to the intracellular face of the cell membrane, which limits the effects of pNaKtide on the membrane-associated (including Na/K-ATPase-associated) c-Src [110,111,112]. Other than this (at least partial) specificity, pNaKtide affects the basal Src activity much less than that of the specific c-Src inhibitor PP2 (which is a very promising factor that will not eliminate the normal cellular c-Src function), and does not affect IGF-induced ERK activation in cardiac myocytes. Furthermore, pNaKtide is effective in disrupting the formation of the Na/K-ATPase/Src receptor complex in a dose-dependent manner. Furthermore, pNaKtide blocks the ouabain-induced activation of c-Src and ERK1/2 and hypertrophic growth in cardiac myocytes. Needless to say, it is imperative to further investigate the underlying mechanism(s) in which (p)NaKtide controls redox-sensitive Na/K-ATPase signaling and systemic redox status in cells, tissues, and whole bodies.

## Abbreviations

CKD	chronic kidney disease
CTS	cardiotonic steroids
IP3Rs	inositol 1,4,5-trisphosphate receptors
MBG	marinobufagenin
NCX	Na^+^/Ca^2+^ exchanger
PLC-γ	phospholipase C–γ
PNx	partial (5/6^th^) nephrectomy
RNS	reactive nitrogen species
ROS	reactive oxygen species
